# The beta-adrenergic agonist salbutamol modulates neuromuscular junction formation in zebrafish models of human myasthenic syndromes

**DOI:** 10.1093/hmg/ddy062

**Published:** 2018-02-16

**Authors:** Grace McMacken, Dan Cox, Andreas Roos, Juliane Müller, Roger Whittaker, Hanns Lochmüller

**Affiliations:** 1 Institute of Genetic Medicine, The John Walton Muscular Dystrophy Research Centre, Newcastle University, International Centre for Life, Newcastle Upon Tyne NE1 3BZ, UK; 2 Tissue Omics Project Group, Biomedical Research Department, Leibniz-Institut für Analytische Wissenschaften-ISAS-e.V., 44227 Dortmund, Germany; 3 Institute of Genetic Medicine, Wellcome Trust Centre for Mitochondrial Research, Newcastle University, International Centre for Life, Newcastle Upon Tyne NE1 3BZ, UK; 4 Institute of Neuroscience, Newcastle University, Newcastle Upon Tyne NE1 7RU, UK

## Abstract

Inherited defects of the neuromuscular junction (NMJ) comprise an increasingly diverse range of disorders, termed congenital myasthenic syndromes (CMS). Therapies acting on the sympathetic nervous system, including the selective β2 adrenergic agonist salbutamol and the α and β adrenergic agonist ephedrine, have become standard treatment for several types of CMS. However, the mechanism of the therapeutic effect of sympathomimetics in these disorders is not understood. Here, we examined the effect of salbutamol on NMJ development using zebrafish with deficiency of the key postsynaptic proteins Dok-7 and MuSK. Treatment with salbutamol reduced motility defects in zebrafish embryos and larvae. In addition, salbutamol lead to morphological improvement of postsynaptic acetycholine receptor (AChR) clustering and size of synaptic contacts in Dok-7-deficient zebrafish. In MuSK-deficient zebrafish, salbutamol treatment reduced motor axon pathfinding defects and partially restored the formation of aneural prepatterned AChRs. In addition, the effects of salbutamol treatment were prevented by pre-treatment with a selective β2 antagonist. Treatment with the cyclic adenosine monophosphate (cAMP) activator forskolin, replicated the effects of salbutamol treatment. These results suggest that sympathomimetics exert a direct effect on neuromuscular synaptogenesis and do so via β2 adrenoceptors and via a cAMP-dependent pathway.

## Introduction

The development of the neuromuscular junction (NMJ) requires several levels of organization, and occurs in a series of overlapping steps which require interplay between presynaptic nerves and postsynaptic muscle components. NMJs assemble in a narrow central region of the myofibre, where the density of acetylcholine receptors (AChRs) must be high in order to initiate a synaptic potential (1). Prior to innervation, in the step termed prepatterning, clusters of AChRs localize in a central band on muscle fibres (2). Several intrinsic muscle proteins are required for this process including the muscle-specific receptor tyrosine kinase (MuSK) and the low-density lipoprotein receptor-related protein-4 (LRP4) (3,4). Following innervation, some of these prepatterned AChR clusters are actively incorporated into developing NMJs, supporting the view that the postsynaptic apparatus defines its central region for ingrowing motor axons (5). The arriving motor nerve, and the agrin released by it, subsequently stabilize the AChR clusters and cause the dispersal of non-synaptic AChR clusters (6). Agrin binds the preformed MuSK/LRP4 complex to initiate MuSK autophosphorylation and activation, thereby initiating a cascade of signalling pathways leading to AChR clustering and postsynaptic differentiation (7,8). MuSK also recruits the adaptor protein Downstream of Kinase-7 (Dok-7), which stimulates further MuSK phosphorylation (9,10). Thus, the LRP4/MuSK/Dok-7 agrin receptor complex is indispensable for tyrosine phosphorylation and clustering of AChRs.

Components of the LRP4/MuSK/Dok-7 complex are the targets of mutations responsible for subsets of congenital myasthenic syndromes (CMS), which are characterized by fatigable muscle weakness (11). CMS are one of the few neuromuscular diseases for which symptomatic treatments are readily available. For many subtypes, clinical benefit is gained from administration of acetylcholinesterase (AChE) inhibitors, which augment the synaptic response to acetylcholine. For other CMS subtypes, including those with mutations that cause deficits in Dok-7, MuSK, and in end-plate AChE deficiency, the sympathomimetics ephedrine and salbutamol are first-line treatment. These drugs lead to increasing and sustained improvements in muscle strength, with the effect peaking after 3–6 months of commencing treatment (12–14). However, the mechanism by which these drugs may alter neuromuscular transmission in these patients is not known.

In order to address this, we examined the effects of the β2 agonist salbutamol on NMJ development in zebrafish. There are several factors which make zebrafish a useful model system to study synaptogenesis. NMJ development occurs in a similar series of steps in zebrafish as in mammals, with a diffuse elongated band of prepatterned AChRs forming in the central region of adaxial muscle fibres prior to the arrival of the motor growth cone (5). The first NMJs are formed between 16 and 24 h post fertilization (hpf) and by 120 hpf synapses are localized at the end of muscle fibres (the vertical myosepta) and scattered over the entire length of muscle fibres (15,16). The speed at which the neuromuscular system develops has allowed the consequences of deficiency of key synaptic proteins which would be lethal in mammals to be examined in zebrafish before death occurs, including functional knockouts of AChRs, AChE, MuSK, Rapsyn and Dok-7 (17–21).

Here, we use antisense morpholino oligonucleotides (MO) which knockdown expression of two key postsynaptic proteins, Dok-7, which leads to impaired motility and smaller NMJs, and *unplugged*, the zebrafish orthologue of MuSK, which demonstrates aberrant motor axon pathfinding and impaired AChR prepatterning (19,21,22). We show that salbutamol leads to improvement in AChR clustering, motor axon guidance and the development of prepatterned AChR clusters, as well as having functional benefit on motility and swim behaviour in zebrafish embryos. In addition, we show that the primary effect of salbutamol on NMJ development is mediated via β2 receptors and via the cyclic adenosine monophosphate (cAMP)/protein kinase A (PKA) pathway. Our results suggest that β2 agonists directly influence synaptic organization and that their therapeutic benefit in myasthenic disorders may be through morphological restoration of the NMJ.

## Results

### Salbutamol treatment of Dok-7 morphant zebrafish improves motility and synaptogenesis

**Figure 1.**
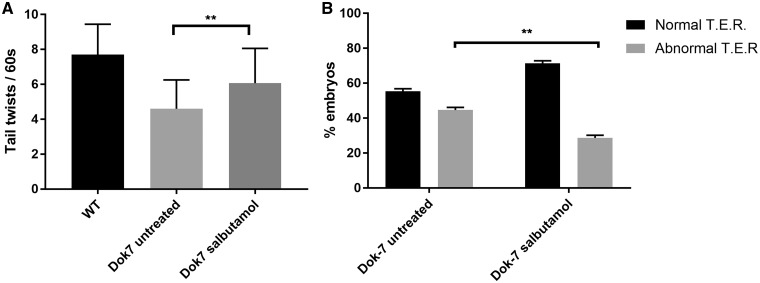
Dok7 morphant zebrafish demonstrate improved motility in the presence of salbutamol. (**A**) When monitored at 24 hpf, Dok7 MO-injected embryos are able to perform tail twisting movements in their chorion but do so at lower frequency than WT embryos. Following salbutamol treatment, Dok7 embryos performed tail twists at an increased frequency (mean 6.1 per 60 s) compared with untreated embryos (mean 4.6 per 60 s). A total of 30 embryos were observed for each category. ** indicates *P* < 0.01, Student’s *t*-test, *n* = 30 treated and 30 untreated embyros observed. (**B**) Dok7 embryos demonstrate equal percentages of normal and abnormal touch-evoke response (TER) to stimuli at 48 hpf. Embryos treated with salbutamol demonstrate an increased percentage of normal swim behaviour. ** indicates *P* < 0.01, chi-square test, *n* = 3 tests on 30 embryos were performed for each category. All error bars depict S.E.M.

In zebrafish, knockdown of NMJ proteins usually affects motility and swimming behaviour. The first locomotor behaviour stage observed is spontaneous alternating tail movements inside the chorion from 17 hpf. Dok-7 MO-injected embryos perform these at a reduced frequency to wild-type (WT) embryos, when measured at 24 hpf (21). Following 24 h of salbutamol treatment, Dok-7 embryos performed these tail twists at an increased frequency compared with untreated Dok-7 embryos (Fig. 1A, *P* < 0.01). After hatching from the chorion, zebrafish embryos respond to touch stimulus rapidly by swimming away from the stimulus. Approximately 50% of Dok-7 embryos demonstrate an abnormal response to touch stimuli, and may make abnormal twitching movements or fail to move away from the stimulus (21). Following salbutamol treatment, an increased percentage of Dok-7 embryos demonstrated normal swimming behaviour following touch stimuli (Fig. 1B, *P* < 0.01).

**Figure 2.**
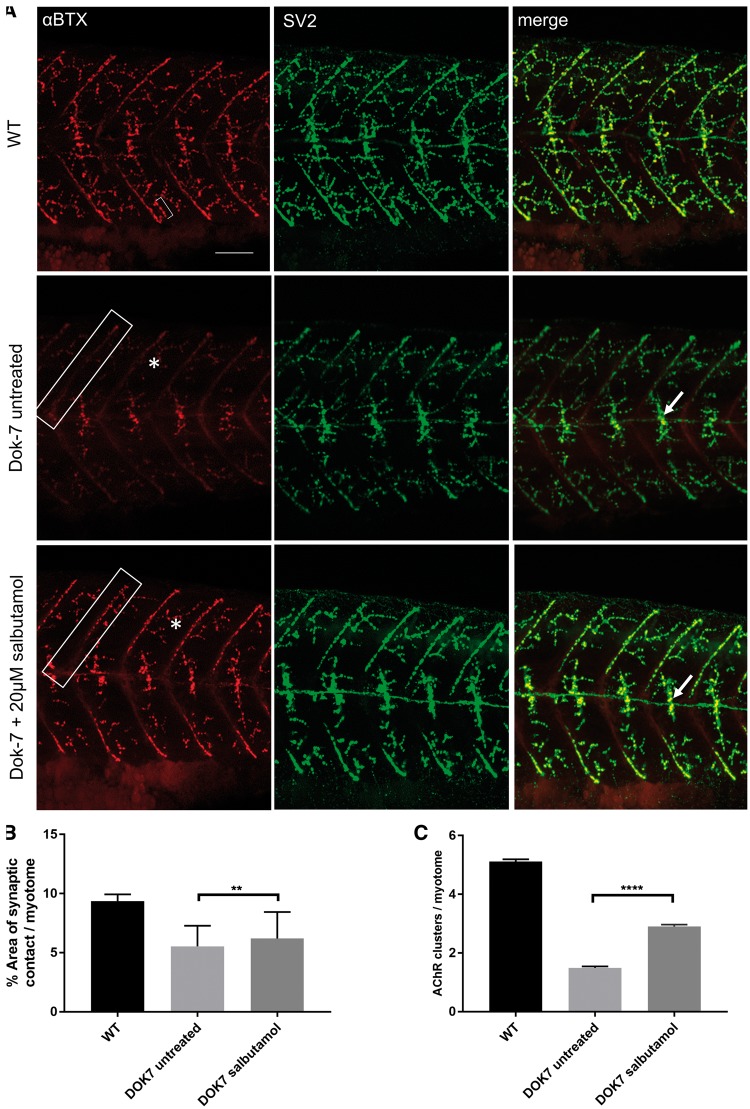
Salbutamol treatment of Dok-7 MO-injected zebrafish improves NMJ morphology. Lateral views of 48 hpf embryos with neuromuscular synapses labelled with antibodies against SV2 (green, presynaptic vesicles) and αBTX (red, postsynaptic AChRs). Scale bar = 50 µm. (**A**) In Dok7 MO-injected embryos, the focal innervation point along the horizontal myoseptum is reduced in size compared with wild type, but is increased following treatment with 20 µm salbutamol (arrows). In addition, the morphology of myoseptal (boxes) and myotomal (asterisks) AChR clusters is partially rescued with salbutamol treatment. A representative 20 μm^2^ AChR cluster is labelled with a bracket. (**B**) Following salbutamol treatment, the area of synaptic contact on the horizontal myoseptum is significantly increased (** indicates *P* < 0.01, Student’s *t*-test, *n* = 160 treated and 160 untreated myotomal segments examined). (**C**) Salbutamol treatment caused a significant increase in the number of AChR clusters >20 μm^2^ per myotome in Dok7 zebrafish embryos (**** indicates *P* < 0.0001, Student’s *t*-test, *n* = 100 treated and 100 untreated myotomal segments examined).

The morphology of the NMJ was examined in the Dok-7 zebrafish by immunostaining of AChRs and presynaptic nerve terminals (Fig. 2A). Although Dok-7 zebrafish lack prepatterned AChR clusters, they do subsequently develop AChR clusters opposed to the growth cone along the horizontal midline of each myotome (the horizontal myoseptum). In Dok-7 embryos, this area of pre- and postsynaptic co-localization is smaller than in WT embryos. We calculated the area of co-localization following salbutamol treatment as a percentage of the area of the myotome it occupied. Following salbutamol treatment, the area of co-localization on the horizontal myoseptum increased significantly (Fig. 2B, 6.2% in treated Dok-7 embryos, compared with 5.5% in untreated Dok-7 embryos, *P* < 0.01). In addition, the number of AChR clusters larger than 20 μm^2^ on myotomal muscle fibres increased following salbutamol treatment (Fig. 2C, mean number of AChR clusters per myotome 2.9 in treated Dok-7 embryos, compared with 1.49 in untreated Dok-7 embryos, *P* < 0.0001).

### Salbutamol treatment alleviates axon pathfinding defects in zMuSK zebrafish

The results of salbutamol treatment in the Dok-7 zebrafish prompted us to explore the effect of salbutamol on NMJ development when another key postsynaptic protein was knocked down. We studied the effect of salbutamol in *unplugged* morphants, the zebrafish homologue of MuSK. *Unplugged* has two splice variants; splice variant 1 (SV1) which has a role in AChR prepatterning and axon guidance, and splice variant 2 (full-length, FL) which is dispensable for both of these processes (19,22). We used a MO targeting only the *unplugged* SV1 isoform (19). *Unplugged* SV1 transcripts are first detectable at 10 hpf and expression is restricted to adaxial muscle cells until late somitogenesis (>24 hpf) when it is downregulated to coincide with the arrival of motor growth cones and lateral migration of adaxial cells, thus embryos were examined in the first 24 hpf (19). We refer to zebrafish embryos with MO knockdown of *unplugged* SV1 as zMuSK.

In zebrafish, each myotomal muscle is initially innervated by just three primary motor neurons, termed CaP, MiP and RoP (16). Initially, all three motor growth cones extend along the medial surface toward a ‘choice point’ on the horizontal myoseptum (16,23). Here, growth cones pause and make contacts with muscle cells, and then select a specific path to ventral, dorsal or medial myotomal regions.

**Figure 3.**
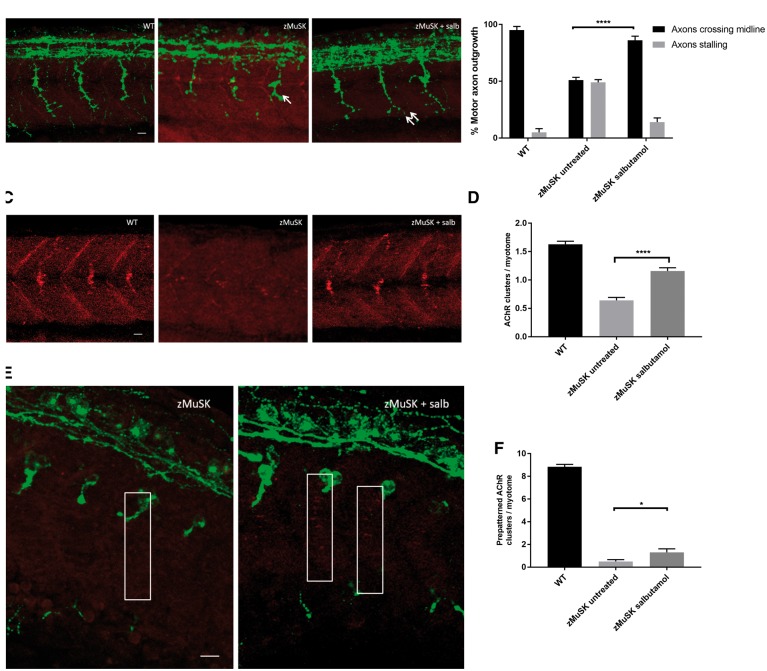
Salbutamol treatment of zMuSK MO-injected embryos improves motor axon pathfinding defects and AChR clustering. Lateral views of embryos with neuromuscular synapses labelled with antibodies against SV2 (green, presynaptic vesicles) and αBTX (red, postsynaptic AChRs). Scale bars = 10 µm. (**A**) zMuSK MO-injected embryos at 24 hpf demonstrate characteristic stalling of the outgrowing motor axon at the choice point on the horizontal midline (arrow). Following salbutamol treatment more axons cross the horizontal midline and extend to the periphery of the myotome (double arrow). (**B**) Quantification of the improvement of motor axon growth following salbutamol treatment. (**** indicates *P* < 0.0001, chi-square test, *n* = 240 treated and 240 untreated myotomal segments examined). (**C**) zMuSK embryos at 24 hpf display almost complete absence of AChR clustering. In salbutamol-treated zMuSK embryos, AChR clustering is restored. (**D**) Quantification of the improvement in AChR clustering following salbutamol treatment of zMuSK embryos. AChR clustering is significantly increased with the mean number of AChR clusters >20 μm^2^ per myotome 1.16 in salbutamol treated embryos compared with 0.64 in untreated embryos (**** indicates *P* < 0.0001, Student’s *t*-test, *n* = 100 treated and 100 untreated myotomal segments examined). (**E**) zMuSK MO-injected embryos at 17 hpf. Untreated embryos lack elongated, diffuse prepatterned clusters on the myotomal surface prior to the arrival of the motor growth cone (boxed areas), but following salbutamol treatment some zMuSK embryos display prepatterned clusters. (**F**) Quantification of the effect of salbutamol on prepatterned AChR clustering in zMuSK embryos. Following salbutamol treatment, the number of prepatterned AChR clusters >3 μm^2^ in zMuSK embryos increased, with a mean of 0.5 prepatterned clusters per myotome in untreated embryos, compared with 1.3 in salbutamol-treated embryos (* indicates *P* < 0.05, Student’s *t-*test, *n* = 30 untreated and 30 untreated myotomal segments examined).

In contrast to WT embryos, zMuSK embryos display characteristic stalling and branching at the choice point, even though their dorsal adaxial cells are properly specified and migrate correctly (19). Following treatment with salbutamol, zMuSK MO-injected embryos displayed a restoration of axonal pathfinding defects at 24 hpf (Fig. 3A and B); in untreated embryos, 51% (*n* = 123/240) of imaged somites displayed axons which failed to cross the midline at 24 hpf, compared with 14% (*n* = 34/240) in embryos treated with salbutamol (*P* < 0.0001). In addition, zMuSK knockdown causes complete absence or greatly reduced numbers of AChRs at 24 hpf. In zMuSK MO embryos treated with salbutamol, however, AChR clusters could be seen as a band on the horizontal myotome at 24 hpf (Fig. 3C and D), which was similar although not as organized as the band seen in WT embryos.

In order to ascertain whether salbutamol treatment had an effect on the formation of prepatterned AChR clusters, we examined the caudal segments of embryos at 17 hpf for the presence of AChR clusters prior to the arrival of the motor growth cone. In salbutamol-treated embryos, a partial rescue of the formation of prepatterned AChR cluster formation was observed (Fig. 3E and F).

### The effects of salbutamol on NMJ development are via the β2 receptor

**Figure 4.**
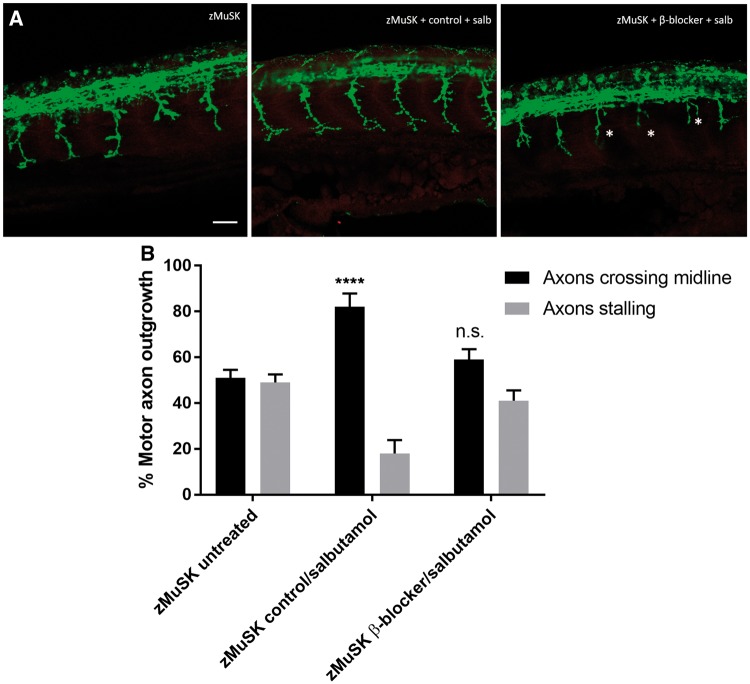
Pre-treatment with a selective β2 antagonist blocks the rescue of NMJ development by salbutamol. Lateral views of 24 hpf embryos with neuromuscular synapses labelled with antibodies against SV2 (green, presynaptic vesicles) and αBTX (red, postsynaptic AChRs). Scale bar** **= 50 µm. zMuSK embryos were treated with 5 µm of the selective β2 antagonist ICI118, 551 or control (water) added to E3 medium for 3 h prior to the addition of 20 µm salbutamol. (**A**) In zebrafish embryos pre-treated with ICI118, 551 no rescue of the axonal pathfinding defects was seen following 21 h of salbutamol treatment, with stalling and branching of motor growth cones (asterisks). (**B**) Quantification of the effect of pre-treatment of ICI118, 551 on motor axon growth. Following pre-treatment with 5 µm ICI118, 551, salbutamol treatment did not have an effect on axon pathfinding (n.s. *P* > 0.05, ****P < 0.0001, chi-square test, *n* = 240 treated and 240 untreated myotomal segments examined).

In order to ascertain whether the effects seen following salbutamol treatment were due to its action as a β2 agonist, or via an off-target effect, we pre-treated zebrafish embryos with the selective β2 antagonist ICI118, 551. Following 3 h of pre-treatment with this β2 blocker, the addition of salbutamol failed to have the same effect on motor axon growth cones (Fig. 4), indicating that the effects observed are mediated via β2 receptors.

### The effects of salbutamol on NMJ development are via the cAMP signalling pathway

**Figure 5.**
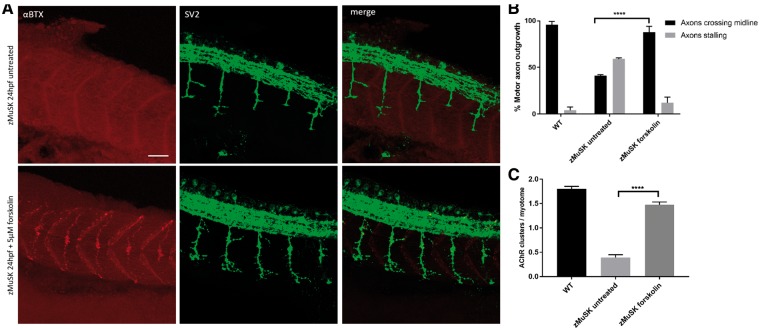
The effects of β2 receptor agonist activation on NMJ morphology are replicated by cAMP activation. Lateral views of 24 hpf embryos with neuromuscular synapses labelled with antibodies against SV2 (green, presynaptic vesicles) and αBTX (red, postsynaptic AChRs). Scale bar = 50 µm. (**A**) zMuSK embryos at 24 hpf demonstrate improvement in AChR clustering and axon pathfinding defects following forskolin treatment. (**B**) Quantification of motor axon guidance defects. About 88% (*n* = 28/240) of imaged somites of zMuSK embryos treated with forskolin displayed no axon guidance defects, compared with 41% (*n* = 142/240) of those in untreated zMuSK embryos. **** indicates *P* < 0.0001, chi-square test. (**C**) The number of AChR clusters larger than 20 μm^2^ on myotomal muscle fibres increased with forskolin treatment, with mean number of clusters 0.39 per myotome in untreated embryos, and 1.48 in treated zMuSK embryos. **** indicates *P* < 0.0001, Student’s *t-*test, *n* = 100 treated and 100 untreated myotomes examined.

Agonist activation of β2 adrenoceptors stimulates a cascade of intracellular signalling pathways, with the most prolific being the cAMP/PKA pathway. Following β2-adrenoceptor stimulation, the receptor couples to the Gαs subunit and activates adenylate cyclase, generating cAMP. However, several additional pathways which are Gαs-independent can also be activated by β2-adrenoceptors binding (24). In order to ascertain whether the downstream signalling pathway involved in rescue of the NMJ morphology in zebrafish was via cAMP or via a Gαs-independent pathway, we treated zebrafish embryos with the adenylyl cyclase activator forskolin. Following incubation for 24 h with 5μM forskolin, the effects of salbutamol axon pathfinding (Fig. 5A and B) and AChR clustering (Fig. 5C) in zMuSK embryos were replicated. In addition, forskolin treatment lead to an increase in the number of prepatterned AChR clusters (mean 3.5 prepatterned clusters per myotome in forskolin-treated embryos, compared with 0.5 in untreated embryos, *P* < 0.0001).

## Discussion

The process of neuromuscular transmission is complex and involves several levels of specialization. There are numerous cellular and molecular pathways which may be potential pathological or therapeutic targets. Given that β_2_ adrenoceptors activate many signalling pathways linked to a variety of changes in different tissues and cell types, the mechanisms of improved neurotransmission by β-agonists may be numerous, and may include postsynaptic expansion of end plates, growth of presynaptic nerve terminals or restoration of normal levels of neurotransmitter release. Zebrafish models allow the analysis of the effect of β-agonists during each step of NMJ development, which in turn may give some indication as to which molecular mechanisms may be involved, based on the previously characterized role of key NMJ proteins at each of these steps. We observed that salbutamol treatment led to morphological improvement of the NMJ at several developmental steps: an increase in the area of co-localization of presynaptic nerve terminals and AChR clusters on the horizontal myoseptum in Dok-7 MO-injected embryos, improvement in AChR clustering at in Dok-7 MO-injected embryos, rescue of the axonal pathfinding defects in zMuSK MO-injected embryos and improvement in the formation of prepatterned AChR clusters. We did not observe an improvement in the number of prepatterned AChR in Dok-7-deficient zebrafish (data not shown). This is unexpected, given the known function of the MuSK/Dok-7 complex at the NMJ, and it is possible that changes in expression of prepatterned AChR in the Dok-7 MO-injected embryos may have been too small to be detected as changes on immunostaining. In addition, we observed that the adenylyl cyclase activator forskolin, lead to dramatic improvements in axonal pathfinding and AChR clustering, and improved AChR prepatterning in zMuSK zebrafish.

Each of these steps are governed primarily by muscle-derived signals in zebrafish (19,25). This is in-keeping with the fact that it is mainly postsynaptic CMS subtypes in which the effect of β-agonist therapy is beneficial. Salbutamol treatment leads to an increasing and sustained response in Dok-7 CMS (13). Although the pathomechanisms of Dok-7 CMS are not yet fully understood, it is widely accepted that mutations in *DOK7* impair Dok-7’s ability to activate MuSK. This assumption is supported by the fact that many mutations in Dok-7 CMS target the COOH terminal, phosphotyrosine binding (PTB) or pleckstrin homology (PH) domains, which are critical sites for activation of MuSK *in vitro* and *in vivo* (26,27). The other CMS subtype in which treatment with β agonists leads to consistent improvement is CMS due to end-plate AChE deficiency, which is caused by mutations in *COLQ* (28,29). In addition to its role in anchoring AChE in the basal lamina, the C-terminus domain of ColQ binds MuSK, and ColQ has been shown to have an important regulatory role in postsynaptic differentiation through this interaction (30). The pathology of Dok-7 and ColQ CMS subtypes may be unified by the role of both proteins in postsynaptic specialization and AChR clustering. β agonist therapy may therefore also play a role in postsynaptic differentiation in mammals. Indeed, several lines of evidence point toward cAMP/PKA-dependent pathways involved in metabolic stabilization of AChRs (31–33).

The effect of salbutamol on both axonal pathfinding and number of prepatterned AChR clusters in zebrafish indicates a possible role for β2 agonists in upregulation or activation of a MuSK-dependent pathway in early synapse development, which has been previously shown to be indispensable for normal regulation of both of these processes in zebrafish (19). There are several potential pathways by which β2-adrenergic agonist-mediated activation of the cAMP/PKA pathway could be activating MuSK, including the β-catenin/Wnt signalling pathway. The zebrafish homologue of MuSK has been previously shown to interact with Wnt11r to restrict AChR prepattern in a central muscle zone (22), in a similar manner to the interaction with Wnt signalling and mammalian MuSK (34). However, given that salbutamol and forskolin treatment led to only partial rescue of the motility and NMJ defects in our zebrafish models, it is conceivable that additional pathways not upregulated by cAMP signalling are also involved.

The effects of forskolin on the NMJ not only indicate the cAMP mimetic action of salbutamol at the NMJ, but also were even more marked than those observed following salbutamol exposure. This more obvious effect may reflect increased absorption, bioavailability or efficacy of forskolin compared with salbutamol in zebrafish. Forskolin has been used in several clinical settings including asthma, congestive cardiomyopathy and obesity (35–37). However, its efficacy in neuromuscular disease is unknown. One possible role for forskolin could be in the treatment of CMS patients who have become tolerant to the effect of beta-adrenergic receptor stimulation following many years of treatment, given that its mechanism of action is receptor independent. Further preclinical studies will be required to confirm these findings.

We accept there are limitations to our disease models. Whilst treatment of zebrafish embryos provides a model for NMJ formation and exploring the role of β2 agonists in this process, effects can only be studied over a short treatment period. The therapeutic effect of β agonists seen in CMS patients is a delayed one, with an increasingly positive response after many months of treatment (13). The relatively short exposure duration of the zebrafish to salbutamol and forskolin may further explain why only a partial rescue of the phenotype was observed in both models. In addition, patients with CMS due to *DOK7* mutations frequently present after infancy or in early adulthood (38), and postnatal knockdown of Dok-7 gene expression has been shown to cause CMS in mice (39), indicating a further role for Dok-7-mediated activation of MuSK in postnatal NMJ maintenance. Therefore, it is possible that the improvement seen in β-agonist-treated CMS patients is due to an additional role they play in NMJ homeostasis. This hypothesis is supported by the recent finding of sympathetic innervation in close contact with the NMJ contributing toward NMJ maintenance (32). Further work will be in identifying the mechanism of altered postsynaptic specialization by either endogenous or exogenous β2-adrenoceptor ligands and cAMP activation. Nevertheless, the effects demonstrated during NMJ development in these zebrafish models, indicate that sympathomimetics may alter the regulation of key NMJ signalling molecules, and suggest that β2 agonists illicit their therapeutic effect in myasthenic disorders through a direct effect at the NMJ.

## Materials and Methods

### Zebrafish husbandry

The Golden strain (slc24a5^b1/+^) of zebrafish was used (Zebrafish International Resource Centre, Oregon). Zebrafish embryos were raised in E3 medium (5 mm NaCl, 0.17 mm KCl, 0.33 mm CaCl_2_, 0.33 mm MgSO_4_, 0.01% methylene blue) at 28.5°C and staged in hours post fertilization (hpf) according to standard protocols.

### Phenotype and motility observation

Tail twists in chorion at 24 hpf were measured by observing each embryo for 60 s and counting total number of complete twists. A total 30 embryos were measured for each category. Touch-evoked swimming response (TER) was observed by touching the head or tail of the zebrafish with a fine pipette tip. TER was defined as abnormal if embryos exhibited either circling movements, twitching with no movement away from the stimulus, or had no movement at all as a response. At least 20 embryos were observed for each category and the experiment was repeated five times.

### Antisense morpholino oligonucleotide knockdown

Antisense MO were injected into the yolk of one-to-two cell stage embryos. Dok-7 embryos were injected with 15 ng of Dok-7 MO. MuSK embryos were injected with 5 ng of zMuSK MO. In addition, control MOs were injected at the same concentration for each injection experiment. MOs were purchased from Gene Tools LLC (Pilomath, OR). A splice-blocking MO was used to target Dok-7 transcripts at exon 2: 5′-ATTTATAGGATTTACCTGCTACCGG. This splice-blocking MO causes skipping of the exon and premature translation termination through targeting of the splice donor site of exon 2 (21). For MuSK experiments, a MO targeting the unplugged splice transcript variant 1 (unplugged/SV1) was used (19,22): 5′-GTAGAGGATTACCGTATTGCCGTT. This causes skipping of exon 2, frameshift and a premature stop codon after 24 cryptic residues. The Gene Tools standard control MO (5′-CCTCTTACCTCAGTTACAATTTATA-3′) targeting a human beta-haemoglobin gene was used as a negative control. MOs were suspended in 1× Danieau buffer (58 mm NaCl, 0.7 mm KCl, 0.4 mm MgSO4, 0.6 mm Ca(NO_3_)_2_, 5 mm HEPES; pH 7.6) with phenol red as an injection indicator. At least two independent MO injection experiments were performed for each MO and 200–800 injected embryos were evaluated for each treatment regimen.

### Treatment of zebrafish with salbutamol, ICI118, 551 and forskolin

Zebrafish embryos were raised post injection in E3 medium containing the compounds or control vehicle. We conducted preliminary dose response testing for salbutamol (1, 10, 20 and 50 µm), forskolin (0.1, 1, 3, 5 and 10 µm) and ICI118, 551 (1, 3, 5, 20 and 50 µm). Concentrations of 20 µm salbutamol, 5 µm forskolin and 5 µm ICI118, 551 resulted in strong effects and limited toxicity, and these concentrations were selected for all further experiments. For salbutamol treatment, embryos were exposed to 20 µm concentration of salbutamol (Sigma) or 20 µm of methanol for up to 72 hpf. For pre-treatment with the selective β2 receptor blocker ICI118, 551, zebrafish embryos were incubated with E3 medium containing either 5 µm of ICI118, 551 hydrochloride (abcam) or 5 µm of distilled water. After 3 h, 20 µm of salbutamol was added to the same E3 medium. For forskolin treatment, zebrafish embryos were raised in E3 medium containing 5 µm of forskolin (abcam) or 5 µm ethanol for up to 72 hpf.

### Zebrafish whole-mount immunofluorescence staining

Embryos were fixed in 4% paraformaldehyde (PFA) in phosphate-buffered saline (PBS) overnight and then permeabilized in cold acetone at −20°C. Depending on their age, 3–5 days post fertilization old larvae were permeabilized with collagenase A (Roche Diagnostics, 1 mg/ml) for 60 min. Embryos were blocked in 5% horse serum in PBS 0.1% Tween-20 (PBS-T). Embryos were incubated in 5% horse serum in PBS-T containing primary antibody overnight at 4°C (presynaptic nerve terminals: SV2, 1: 200 from Developmental Studies Hybridoma Bank, Iowa), washed several times with PBS-T and incubated with secondary antibody (goat anti-mouse Alexa Fluor 488, donkey anti-mouse Alexa Fluor 594, goat ant-rabbit Alexa Fluor 680, all Invitrogen). AChRs were visualized by using Alexa Fluor 594-conjugated α-bungarotoxin (1 μg/ml, Invitrogen). Immunofluorescence staining was imaged using a Nikon confocal microscope (Nikon A1R Invert). For quantification of NMJ morphological changes, 30 embryos were imaged for each category. Because of the rostral-to-caudal gradient of embryonic development in zebrafish, we used the same myotomal segments for comparison, by examining the five myotomes centred around the caudal-most part of the yolk extension. WT, untreated morphant and treated morphant tissues were processed for immunofluorescence in parallel and images were acquired with the same confocal microscopy settings (laser power, gain, magnification and Z-stack interval). Image analysis was performed using ImageJ software. Immunostained structures were counted as NMJs when SV2 was opposed to α-bungarotoxin with at least 50% co-localization. The outer perimeter of each area of co-localization on the horizontal myoseptum was drawn by hand and the enclosed area was measured. The area of each myotome was also measured, and the area of co-localization was measured as a fraction of the myotome it occupied. For AChR quantification, AChR clusters were thresholded and their area and intensity measured, and the number AChR clusters greater than 20 μm^2^ in size per myotome were compared. For prepatterned AChR quantification, the five caudal-most myotomal segments were compared in 17 hpf embryos, and the number of AChRs greater than 3 μm^2^ per myotome were quantified. Axon pathfinding defects were quantified by counting the percentage of myotomes with axons which failed to cross the horizontal midline at 24 hpf. When comparing values from treated zebrafish with values from untreated zebrafish an unpaired Student’s *t*-test or chi-square test was used. Thresholds of *P* < 0.05 were considered significant. We confirmed normal distributions of data before performing parametric tests.

*Conflict of Interest statement* **.** None declared. 
